# Libman-Sacks endocarditis in a child with systemic lupus erythematosus: a case report and literature review

**DOI:** 10.3389/fped.2024.1323943

**Published:** 2024-01-31

**Authors:** Jingyi Lu, Shengfang Bao, Xuemei Xu, Yingying Jin, Chenxi Liu, Yuqi Zhang, Qian Wang, Yanliang Jin

**Affiliations:** ^1^ Department of Rheumatology and Immunology, Shanghai Children’s Medical Center, School of Medicine, Shanghai Jiao Tong University, Shanghai, China; ^2^ Department of Pediatric Cardiology, Shanghai Children’s Medical Center, School of Medicine, Shanghai Jiao Tong University, Shanghai, China; ^3^ Diagnostic Imaging Center, Shanghai Children’s Medical Center, School of Medicine, Shanghai Jiao Tong University, Shanghai, China

**Keywords:** Libman-Sacks endocarditis, systemic lupus erythematosus, immunosuppressive therapy, children, case report

## Abstract

Libman-Sacks endocarditis (LSE) is a cardiac condition characterized by the growth of verrucous vegetation. Although relatively rare in children, LSE is nevertheless a known cardiac manifestation of autoimmune diseases, including systemic lupus erythematosus (SLE). The mitral valve is the most commonly affected region, followed by the aortic valve, while the tricuspid and pulmonary valves are rarely affected. The management of established Libman-Sacks vegetation poses significant challenges, often necessitating surgical interventions, although surgery is not the primary treatment modality. Herein, we present the case of a 14-year-old Chinese female patient whose initial lupus manifestation included LSE, among other symptoms and signs that provided insights into the final diagnosis of SLE. After early comprehensive pharmacological treatment, tricuspid regurgitation and vegetation disappeared within 28 days without necessitating cardiac surgery, indicating that the resolution of LSE vegetation in this patient was achieved through a combination of immunosuppressive and anticoagulant therapy. These findings suggest the potential of this treatment approach as a viable model for the management of LSE in young patients.

## Introduction

Libman-Sacks endocarditis (LSE), also known as non-bacterial thrombotic endocarditis (NBTE), was first described by Libman and Sacks in 1924. It is a typical cardiac manifestation of SLE and/or antiphospholipid syndrome (APS). Pediatric LSE can manifest with various clinical features, including cardiac murmurs, signs of heart failure, embolic events such as stroke or peripheral artery occlusion, as well as constitutional symptoms like fatigue and weight loss (1). Diagnosis of LSE in children requires a high index of suspicion, as symptoms may be subtle or nonspecific. A literature search revealed only 7 reported casesof LSE in children over the past 20 years. The mitral valve is the most frequently affected site in LSE, followed by the aortic valve, while the tricuspid and pulmonary valves are seldom affected (2). Unfortunately, LSE is commonly misdiagnosed as bacterial endocarditis. Herein, we report a case of LSE in a child with a tricuspid valve treated in our department. In this report, we describe the patient's successful treatment course and provide a review of the relevant literature to improve our understanding of the disease, avoid misdiagnosis, and provide evidence for its clinical treatment and prognosis.

## Case presentation

A 14-year-old Chinese female patient experienced hair loss for more than a year. The patient was 160 cm (in the 54th percentile) tall and weighed 49.3 kg (in the 45.8th percentile) (BMI = 19.26). Before being hospitalized at our center, she had experienced a fever of over 38°C, accompanied by persistent cough, for 8 days. In addition, she had experienced chest pain for 6 days and shortness of breath for 3 days. She further experienced chest and back pain, chest tightness, weakness, and an inability to lie down. In addition, the patient experienced pain in the left index finger joint without discoloration and was unable to straighten it. She reported experiencing no night sweats, hemoptysis, or contact history of tuberculosis. Moreover, she had no significant history of high-risk sexual encounters, travel, medical illnesses, or family illnesses. She had been diagnosed with SLE combined with infection at another hospital and was treated with intravenous cefotiam, azithromycin, and piperacillin for 3 days, the exact dosage of which were unknown; however, her symptoms did not improve. No cultures were performed at the other hospital. The patient was subsequently referred to our hospital.

Laboratory tests and imaging were performed upon admission at our hospital. The laboratory data at admission and 6 months after therapy are summarized in Table 1.

**Table 1:** Laboratory data upon admission and at 6 months after therapy.

	Reference range	Admission	After 6 months
White blood cell count (109/L)	4–10	7.83	9.47
Red blood cell count (1,012/L)	4.1–5.3	2.85	4.11
Hemoglobin (g/L)	115–160	56.0	122.0
Platelets (109/L)	100–300	514	342
CRP (mg/L)	<8	140.1	2.2
ESR (mm/h)	0–20	>140	40
IgG(g/L)	7.1–15.6	25.50*	6.17
Albumin-globulin ratio	1.5–2.5	0.7	1.8
Ferritin (ng/ml)	35–55	172.2	39.6
Fibrinogen (g/L)	1.8–3.5	5.55	5.82
APTT(s)	26–36	38.1	29.8
PT(s)	12–14	14.9	10.9
Procalcitonin (ng/ml)	<0.5	0.15	<0.02
Creatinine (µmol/L)	49–90	59	34
24 h urine protein (mg/24 h)	<150	147	61
NT-proBNP (pg/ml)	0–250	1,704	<5
Direct antiglobulin test	<208.4	Positive	Unknown
C3 (g/L)	0.9–1.8	0.73	1.61
C4 (g/L)	0.1–0.4	0.03	0.31
Antinuclear antibody	<1:100	1:10,000	Positive
Anti-dsDNA antibody (IU/ml)	>15	424.02	Negative
Anti-cardiolipin antibody IgA (APL/ML)	<12.0	18.94	2.87
Anti-cardiolipin antibody IgG, A, M (GPL-U/ml)	0–12	20.45	<2.00
Beta2-Glycoprotein I IgA (U/ml)	0–18	21.11	7.58
Beta2-Glycoprotein I IgG (U/ml)	<18	<2.00	<2.00
Anti-U1-nRNP antibody IgG, anti-Smith antibody IgG, anti-SS-A/Ro60kD antibody IgG, anti-SS-A/Ro52kD antibody IgG, anti-SS-B(La) antibody IgG, anti-nucleosome antibody IgG, anti-histone antibody IgG, anti-ribosomal P protein antibody IgG	–	All positive	Anti-U1-nRNP antibody IgG, anti-SS-A/Ro60kD antibody IgG, anti-SS-A/Ro52kD antibody IgG, anti-SS-B(La) antibody IgG are positive

**Figure 1:**
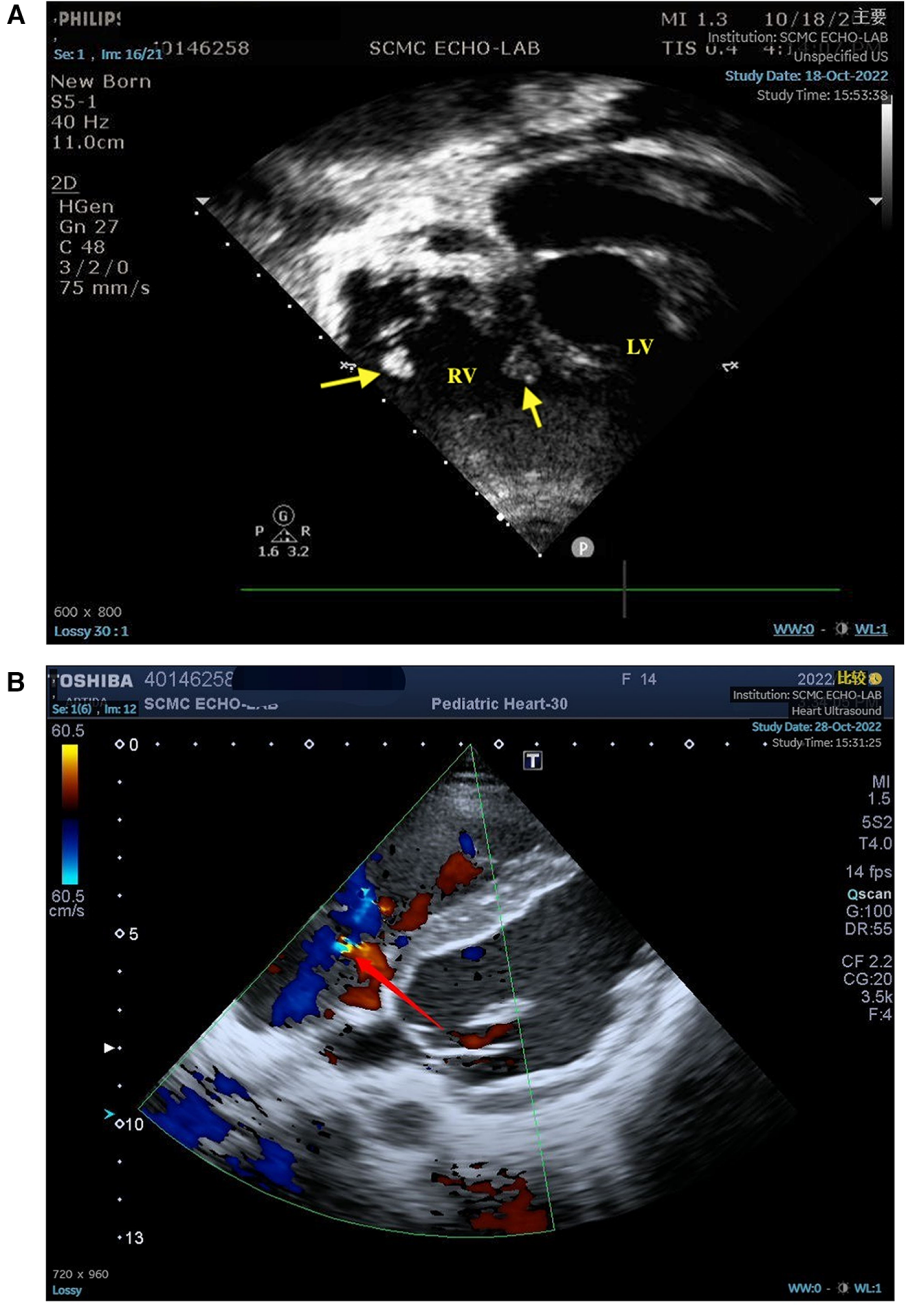
(**A**) Two moderately echogenic masses attached at the level of the tricuspid chordae tendineae measured in atypical apical four-chamber (4C) view. (**B**) The yellow part of the arrow indicates turbulence, indicating tricuspid regurgitation. LV, left ventricle; RV, right ventricle; arrow, masses.

Plain chest computed tomography (CT) revealed bilateral pleural effusions with atelectasis and pericardial effusion, consistent with lung infection. Echocardiography revealed the presence of a small amount of pericardial effusion, pulmonary hypertension, slight thickening of the ventricular septum and left ventricular wall, tricuspid regurgitation, and two moderately echogenic masses attached at the level of the tricuspid chordae tendineae, which oscillated in synchronization with the cardiac cycle and measured approximately 0.82 cm × 0.62 cm, 0.92 cm × 0.86 cm in size (Figure 1). Two blood cultures, one urine culture, one pleural fluid culture, and one respiratory specimen culture were all confirmed to be free of pathogens and fungal growth on day 3. Next-generation sequencing (NGS) of the pleural fluid on day 3 revealed *Enterococcus faecalis* infection. The patient was finally diagnosed with LSE. Cranial magnetic resonance imaging (MRI) revealed multiple abnormal signals in the right frontal and posterior horns of the bilateral ventricles on both sides and bilateral semi-oval centers, SLE-like vascular inflammation in the cerebral vessels on day 10. Peripheral blood smears occasionally showed helmet-shaped red blood cells. The SLEDAI-2 K score 24 h after admission was 22 (6 for serositis and acute pericarditis, 4 for low complement, 4 for autoimmune hemolytic anemia, 6 for anti-Smith antibody positivity, and 2 for alopecia). The patient was in the active stage of the disease and experienced a period of severe disease activity.

**Figure 2:**
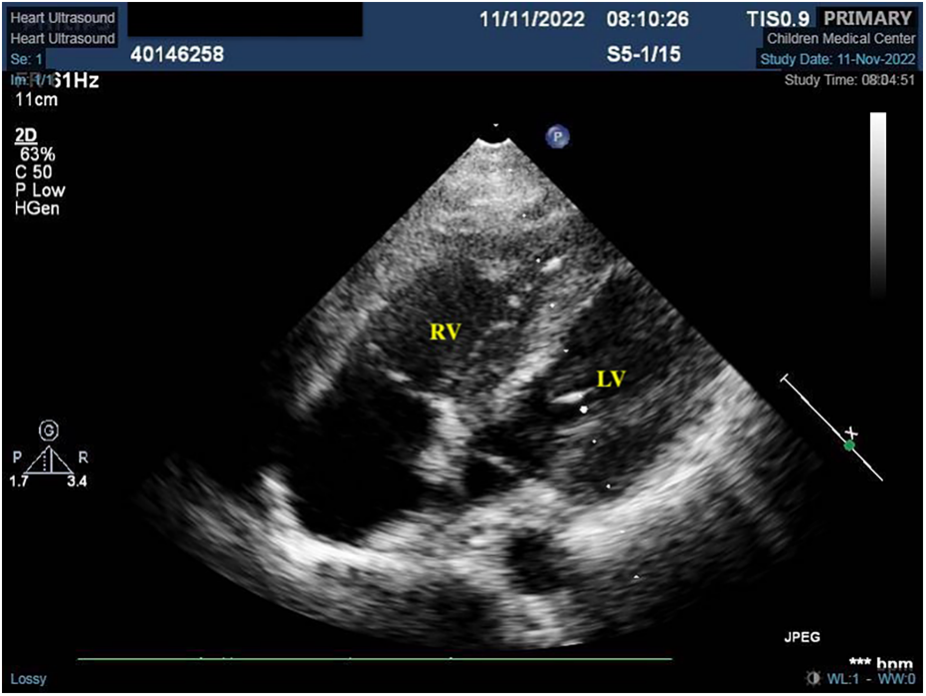
Localized mass disappeared after treatment. *This is an atypical section (that is, not standardized, as it is not standardized because of the need to see the redundant organisms) of the four-chambered heart.

**Figure 3:**
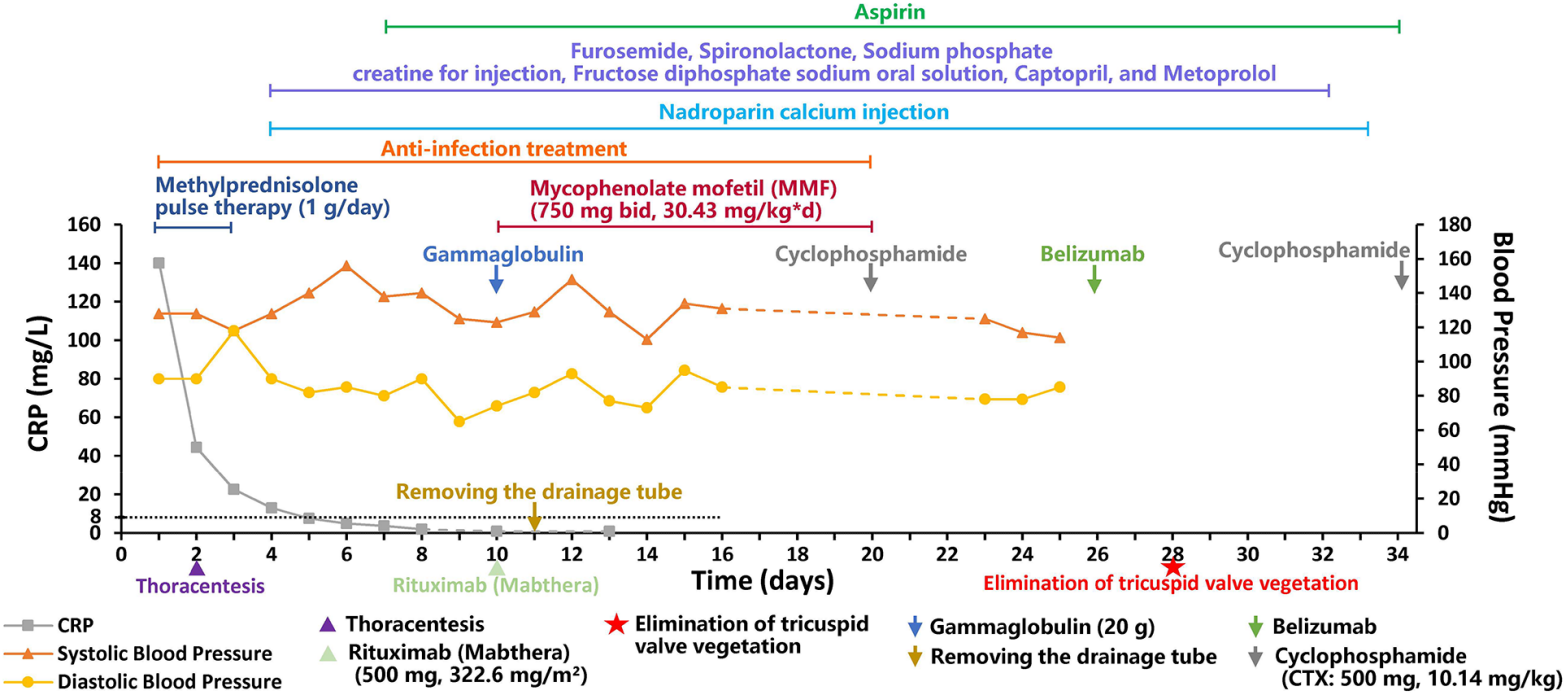
Medication diagram depicting the treatment course.

The patient was finally diagnosed with SLE, lupus encephalopathy, LSE, community-acquired pneumonia (severe), pleural effusion, pericardial effusion (non-inflammatory), severe anemia, and cardiolipin antibody positivity. Anti-infection treatment was administered from day 1 to day 20 of hospitalization in our center. Gammaglobulin (20 g) was also administered on day 10. Fresh frozen plasma was provided as supportive care. The child underwent thoracentesis on day 2 to drain the excess fluid that had accumulated in her chest cavity, and the tube was withdrawn 10 days after drainage. To treat SLE, methylprednisolone pulse therapy was administered for the first 3 days (day 1–3, 1 g/day) of admission, while methylprednisolone (2 mg/kg) was administered from the fourth day. From day 4 to day 32, the patient received furosemide and spironolactone for diuresis, sodium phosphate creatine for injection, fructose diphosphate sodium oral solution for myocardial nutrition, captopril for blood pressure reduction, and metoprolol for heart rate control. On day 10, rituximab (Mabthera) (500 mg, 322.6 mg/m^2^) injection was administered along with mycophenolate mofetil (MMF) (750 mg bid, 30.43 mg/kg*d, day 10-day 20). On day 10, the child's blood pressure was found to be high (148/93 mmHg), and captopril was administered to lower the blood pressure. On day 22, the child's heart rate fluctuated between 128 and 108 bpm during the day, and metoprolol was administered to control the heart rate. The patient was administered low-dose cyclophosphamide (CTX: 500 mg, 10.14 mg/kg) on days 20 and 34 of treatment due to an observed decline in mathematical ability, while cranial MRI revealed SLE-like vascular inflammatory alterations in the cerebral vasculature. On day 26, the patient was administered belizumab (480 mg, 9.74 mg/kg). On day 28, echocardiogram was repeated, showing slight thickening in parts of the tricuspid tendon cords, along with localized echogenicity and elimination of verrucous vegetation (Figure 2). The patient was discharged with a prescription for oral prednisone (60 mg/day) and aspirin (100 mg/day), followed by monthly CTX (1 g, 20.28 mg/kg), and belizumab (480 mg, 9.74 mg/kg) treatment. We created a timeline (Table 2) and medication diagram (Figure 3) depicting the treatment course. A repeat cranial MRI performed 3 months after admission revealed fewer and smaller aberrant signals in the brain compared to the initial MRI. Five months after initial admission, repeat cardiac MRI showed no significant abnormal signals in the cardiac chambers and no significant abnormal myocardial signal, and the SLEDAI-2K score was 0.

**Table 2:** Timeline.

Time	Incidents
3 days age	She had experienced a fever of over 38°C, accompanied by persistent cough, for 8 days.
Admission	The patient was referred to our hospital.
Day 2	The child underwent thoracentesis to drain the excess fluid that had accumulated in her chest cavity, and was treated with anti-infective therapy.
Day 1–3	Methylprednisolone pulse therapy (day 1–3, 1 g/day)
Day 4	Echocardiography revealed slight thickening of the ventricular septum and left ventricular wall, tricuspid regurgitation and tricuspid vegetations
Day 10	Cranial MRI revealed multiple abnormal signals and SLE-like vascular inflammation in the cerebral vessels.
Day 10	Immunosuppressive therapy was initiated for SLE.
Day 28	A repeat echocardiographic examination revealed the elimination of verrucous vegetation.

## Discussion

LSE is rare in children. Patients with antiphospholipid, anti-ro/SS-A, and anti-la/SS-B antibody-positive SLE are more likely to have valve involvement than those without (3, 4). Patients with LSE are typically asymptomatic unless the lesions progress to more severe valvular dysfunction or embolic events, which may be related to highly variable SLE activity (alternating between exacerbations and remissions) and standard anti-inflammatory and/or anti-thrombotic therapy (1). Life-threatening manifestations, such as cardiac tamponade and cardiogenic shock, may present as the initial symptoms (5). The pathogenesis of LSE involves the formation of fibrin-platelet thrombi due to inflammation, which leads to fibrosis and scarring, with subsequent valve dysfunction developing over time (6). LSE is commonly misdiagnosed as infective endocarditis (7). Approximately 10% of patients with infective endocarditis do not exhibit growth in blood cultures, thereby complicating the diagnosis process (8). The vegetations that develop in LSE are more fragile than those that develop in infective endocarditis because of the comparatively diminished *in situ* inflammation, which leads to a greater risk of partial vegetation detachment with resultant embolic phenomena (9).

In our patient, infective endocarditis could not be ruled out because of the persistent fever and increased CRP levels. Multiple pathogen tests returned to normal, and only the presence of enterococcal infection was revealed through NGS analysis in the pleural fluid; however, the fever persisted even after antibiotic therapy in different hospitals. Combined with the patient's laboratory data, we surmised that the persistent fever and elevated CRP levels may be due to SLE activity. In one prior study, LSE was identified as an independent risk factor for neuropsychiatric lupus, which increases the risk of cerebral thrombosis, stroke, transient ischemia, and neuropsychiatric symptoms in patients with LSE (10). However, our patient presented with no embolic manifestations other than SLE-like vascular inflammatory changes on cranial MRI with diminished in mathematical ability. This indicates that the vegetation was lysed following treatment.

In this case, MMF, rituximab, CTX, and belizumab were administered as immunosuppressants. According to the “2019 EULAR Recommendations for the Management of Systemic Lupus Erythematosus" (11), MMF is a potent immunosuppressant that has demonstrated efficacy in both renal and non-renal lupus, although it may not be effective in treating neuropsychiatric manifestations of the disease. In cases of persistent or recurrent extrarenal disease activity, the addition of belimumab should be considered. In situations where the disease poses a threat to vital organs and is refractory to other treatments, rituximab may be a viable option. It is crucial to initiate high-intensity immunosuppressive therapy during the induction phase to effectively manage disease activity in severe cases of SLE. So, in this case, rituximab and MMF were administered together on day 10. Belimumab is a recombinant fully humanized IgG1*λ* monoclonal antibody that targets and inhibits B-cell activating factor (BAFF), also known as B lymphocyte stimulator (BLyS) (12). It reduces the differentiation of B-cells into plasma cells that produce immunoglobulins, decreases serum autoantibodies, and continues to reduce immune complex deposition, thereby delaying organ damage and overall improving lupus outcomes.

In the past 20 years, only 7 cases of pediatric LSE have been documented. The clinical data of these cases and our case, totaling 8 children, are summarized in Table 3.

**Table 3:** Clinical data of 8 cases of SLE combined with LSE in children.

Author	Age/Sex	APS	Heart changes	Treatment	Prognosis
Sharma et al. (2)	14/female	Yes	Mitral valve hemi-thickening	Mitral valve repair, steroids and intermittent immunosuppressive therapy	10 years of follow-up, mild mitral valve insufficiency, lupus nephritis, APS
Kohler et al. (13)	16/female	No	Mitral valve vegetation, mitral regurgitation	Aspirin, diuretics, CTX, rituximab	6 months later, the patient had only mild mitral regurgitation, and the size of the vegetation decreased
Kasar et al. (14)	14/female	No	Diffuse thickening of the aortic leaflets, no vegetations, mild aortic regurgitation	Intravenous methyl prednisolone in pulsed doses for 2 days, oral steroids, MMF	Hypertensive (BP 160/100 mm Hg), a decrease in aortic valve thickening
D'Alessandro et al. (15)	12/male	Yes	Mitral valve vegetation, a large aortic valve perforation	Aortic valve repair	1 year of follow-up, mild aortic valve insufficiency
Wałdoch et al. (28)	13/female	Yes	A heterogeneous mass measuring 15 × 10 mm was found adjacent to the septal leaflet of the tricuspid valve	Steroids and anti-coagulants,	Follow-up echocardiography has shown significant regression of the cardiac mass
Riyami et al. (29)	12/female	No	Severe mitral regurgitation with nodular thickening of the valve	Intravenous (IV) methylprednisolone and cyclophosphamide, a tapering dose of oral prednisolone for six months and monthly IV cyclophosphamide and on regular hydroxychloroquine, esomeprazole, calcium and vitamin D	Mitral regurgitation almost resolved in about four months from the initial presentation
Raj (30)	10/female		9 mm × 8 mm thrombus vs. vegetation on echocardiogram	Steroids and Lovenox	The patient's chorea dramatically improved. Heart changes were not mentioned.
This case (2022)	14/female	No	Pericardial effusion, tricuspid regurgitation, left ventricular posterior wall valve tendon cords with proximal annulus vegetation	Hormones, anticoagulation, MMF, rituximab (mabthera), CTX, gammaglobulin, belizumab	28 days of follow-up, disappearance of vegetations; 5 months of follow-up, no significant abnormal signal in the cardiac chambers and no significant abnormal myocardial signal

Diagnosing LSE can be challenging, especially in the absence of cardiac symptoms. Therefore, it is recommended to implement regular echocardiography screening for patients with SLE, even in the absence of cardiac symptoms (1, 16). Transthoracic echocardiography (TTE) is the first imaging modality of choice for the evaluation of patients with suspected LSE. However, transesophageal echocardiography (TEE) has demonstrated improved sensitivity compared to TTE, for the diagnosis of NBTE. In a recent 20-year cohort study, compared to TTE, TEE was associated with a higher sensitivity for the diagnosis of LSE (17).

The management of LSE in the pediatric age group has not been extensively described. However, there is some evidence available from studies conducted in the adult population. For instance, although corticosteroids can reduce the inflammatory response induced by LSE in adults, they can also increase tissue scarring and fibrosis, which can worsen valve damage (18). However, a case report involving a pregnant woman revealed that following hormone treatment (80 mg/day), the patient experienced a decrease in vegetations and an improvement in ejection fraction (19). Anti-inflammatory and anti-thrombotic therapies have been shown to reduce the activity of SLE, resolve or significantly improve Libman-Sacks vegetation and valve regurgitation, and cause a pathological shift from an active to a mixed or healed type of Libman-Sacks vegetation, which lowers the risk of embolization and may prevent the requirement for high-risk valve surgery (3). According to the 2015 European Society of Cardiology guidelines for the management of infective endocarditis, anticoagulation therapy should be pursued in patients with NBTE if there are no contraindications (20). However, the incidence of thrombotic events in children with antiphospholipid antibody positivity is lower than that in adults, which may be related to the relative difference between the level of blood coagulation protein and development of the vascular endothelium. This suggests that the management and therapeutic strategies for children with LSE and antiphospholipid antibody positivity differ from those in adult patients (21). Furthermore, several adult case studies have previously demonstrated that LSE can be resolved or improved by the administration of immunosuppressive medications in conjunction with anti-thrombotic and hydroxychloroquine therapy (3, 22–26). However, the specific time range during which LSE vegetation can disappear after early active treatment, which requires future multicenter clinical validation, has not yet been documented in the literature.

For severe valve regurgitation, surgery may be considered in patients who show recurrent embolic events despite anticoagulation, large vegetation > 10 mm, and prosthetic valve involvement, depending on the situation (27). In young patients, mitral valve repair, vegetation removal, and ring-shaped plasty are preferred to avoid long-term anticoagulation and promote body growth (2). Artificial valve replacement should be performed when the valve is thickened and tissues are damaged (18).

## Conclusion

In conclusion, LSE is rare in children with SLE. In the present case, resolution of the LSE vegetation and improvement of cardiac function may have been achieved by the early introduction of steroids in conjunction with immunosuppression, anticoagulation, gamma globulin, and plasma support. However, whether this approach can be applied to similar cases with reliable outcomes remains unclear, and further research is necessary to investigate thi aspect of LSE.

## Data Availability

The original contributions presented in the study are included in the article/Supplementary Material, further inquiries can be directed to the corresponding author.
